# Disparities in insecurity, social support, and family relationships in association with poor mental health among US adults during the COVID-19 pandemic

**DOI:** 10.1038/s41598-023-35981-0

**Published:** 2023-06-15

**Authors:** Kexin Zhu, Siyi Wang, Yihua Yue, Beth A. Smith, Zuo-Feng Zhang, Jo L. Freudenheim, Zhongzheng Niu, Joanne Zhang, Ella Smith, Joshua Ye, Ying Cao, Jie Zhang, Dwight A. Hennessy, Lijian Lei, Lina Mu

**Affiliations:** 1 Department of Epidemiology and Environmental Health, School of Public Health and Health Professions, University at Buffalo, The State University of New York, Buffalo, NY USA; 2 Department of Psychology, Vassar College, Poughkeepsie, NY USA; 3 Department of Psychiatry and Pediatrics, University at Buffalo, The State University of New York, Buffalo, NY USA; 4 Department of Epidemiology, Fielding School of Public Health, University of California, Los Angeles, Los Angeles, CA USA; 5 Archer School for Girls, Los Angeles, CA USA; 6 Nichols School, Buffalo, NY USA; 7 The Quarry Lane School, Dublin, CA USA; 8 Department of Population Health Sciences, University of Wisconsin-Madison, Madison, WI USA; 9 Department of Sociology, State University of New York Buffalo State, Buffalo, NY USA; 10 Department of Psychology, State University of New York Buffalo State, Buffalo, NY USA; 11 Department of Epidemiology, School of Public Health, Shanxi Medical University, Taiyuan, Shanxi China

**Keywords:** Public health, Epidemiology, Preventive medicine, Risk factors, Anxiety, Depression, Post-traumatic stress disorder

## Abstract

The COVID-19 pandemic has had a significant impact on mental health. Identifying risk factors and susceptible subgroups will guide efforts to address mental health concerns during the pandemic and long-term management and monitoring after the pandemic. We aimed to examine associations of insecurity (concerns about food, health insurance, and/or money), social support, and change in family relationships with poor mental health and to explore disparities in these associations. An online survey was collected from 3952 US adults between May and August 2020. Symptoms of anxiety, depression, stress, and trauma-related disorders were assessed by the Generalized Anxiety Disorder 7-item scale, the Patient Health Questionnaire-9, the Perceived Stress Scale-4, and the Primary Care Post-Traumatic Stress Disorder Screen, respectively. Social support was measured by the Oslo Social Support Scale. Logistic regression was used and stratified analyses by age, race/ethnicity, and sex were performed. We found a higher prevalence of poor mental health among those who were younger, female, with lower socioeconomic status, and racial/ethnic minorities. Participants who were worried about money, health insurance, or food had higher odds of symptoms of anxiety (OR = 3.74, 95% CI: 3.06–4.56), depression (OR = 3.20, 95% CI: 2.67–3.84), stress (OR = 3.08, 95% CI: 2.67–3.57), and trauma-related disorders (OR = 2.93, 95% CI: 2.42–3.55) compared to those who were not. Compared to poor social support, moderate and strong social support was associated with lower odds of all four symptoms. Participants who had changes in relationships with parents, children, or significant others had worse mental health. Our findings identified groups at higher risk for poor mental health, which offers insights for implementing targeted interventions.

## Introduction

To slow down the transmission of the coronavirus disease-2019 (COVID-19), and to prevent related deaths and illness, the US government issued stay-at-home and lockdown policies as early as March 2020, leading to economic degradation, such as high rate of unemployment and distress due to social isolation, restricted access to food, job insecurity, and financial concerns^1–4^.

Emerging evidence has suggested profound negative consequences of the COVID-19 pandemic on mental health^5–7^. The pandemic crisis and the resulting economic crash have exposed and exacerbated food insecurity^8,9^. From April 23 to May 19, 2020, 23% of households experienced food insecurity, with higher rates observed among  Blacks and Hispanics compared to Whites^10^. Food insecurity caused by the pandemic has been associated with an increased risk of symptoms of anxiety^11^, depression^12^, and stress^13^. Income disruption resulting from unemployment also contributed to poor mental health during the pandemic^4,14^. Unemployment and income disruption can result in the loss of healthcare coverage, leaving individuals and their families without access to essential medical care. This lack of access can result in poor mental health, particularly during widespread illness or public health crises, such as the COVID-19 pandemic. The financial consequences of the pandemic affected racial and ethnic minorities more severely. Between March and May 2020, the unemployment rate increased from 4.4% to 13.2% in the overall population in the United States, from 6.8% to 16.8% among Blacks and from 6.0% to 17.6% among Hispanic or Latinos^15^. However, it is unclear how the impacts of food and finance insecurity on poor mental health differ by race/ethnicity groups.

Previous studies have demonstrated the protective effect of social support on poor mental health in stressful situations^16^. The COVID-19 mitigation strategies, such as social distancing and school closures, placed constraints on social interactions and impeded access to sources of social support^17^. Some studies suggested that greater social support was associated with better mental health during the COVID-pandemic^18^, and a stronger association was found for women and older adults relative to men and younger people^19,20^. In addition, due to increased strain and commotion within households, family relationships have been challenged^21^. However, research on how the change in family relationships might have impacted poor mental health is still limited.

COVID-19-related poor mental health has disproportionately impacted vulnerable populations, such as disadvantaged racial and ethnic groups^22,23^ and younger adults^24,25^. Identifying the groups who might be more severely affected will be informative for preventive strategies and targeted intervention to address concerns on poor mental health during the pandemic as well as long-term management and monitoring after the pandemic. This study aimed to examine associations of insecurity (concerns about money, health insurance, and/or food), social support, and change in family relationships with indicators of poor mental health and to explore if these associations differ by age, race/ethnicity, and sex. We hypothesized that higher insecurity and worse family relationships might be associated with higher odds of poor mental health, and social support may be protective against poor mental health during the pandemic; these associations would vary by age, race/ethnicity, and sex.

## Methods

### Study population

A cross-sectional online survey based on REDCap (Nashville, TN) was conducted between May 13 and August 25, 2020, as previously described^26^. The survey link was distributed via social networks (Twitter and Facebook), email listserv, and ResearchMatch^27^. Adults residing in the US and aged ≥ 18 years were eligible. A total of 4827 individuals answered at least one question in our online survey, and 4140 completed the whole survey (completion rate: 85.8%). We further excluded participants who resided outside of the US (or did not report the country of residence) and those who were under 18 years (or did not report the year of birth), resulting in 3952 participants in our analysis. The study protocol was reviewed by the Institutional Review Board of the University at Buffalo (STUDY00004313) and was determined to be exempt. Informed consent for participation was granted when the voluntary and anonymous survey was completed. The study was carried out in accordance with the approved guidelines.

### Measurement of poor mental health

Symptoms of anxiety, depression, stress, and trauma-related disorders were identified by the Generalized Anxiety Disorder Scale-7 items (GAD-7)^28^, the Patient Health Questionnaire-9 items (PHQ-9)^29^, the Perceived Stress Scale 4 (PSS-4)^30^, and the Primary Care Post-Traumatic Stress Disorder screen (PC-PTSD)^31^, respectively. The GAD-7 consists of seven items that detect the frequency of anxiety symptoms in the past 2 weeks. Each item includes options scored as 0 (not at all), 1 (several days), 2 (over half the days), and 3 (nearly every day), with a total possible score of 21^28^. A total score of ≥ 10 in GAD-7 indicating possible anxiety symptoms achieves a sensitivity of 89% and specificity of 82%^28^. The PHQ-9 consists of nine items scoring the frequency (same as GAD-7) of depressive symptoms over the past two weeks, with a summed score ranging from 0 to 27^29^. The PHQ-9 is a reliable and robust instrument for screening depressive symptoms in adults, with a sensitivity of 88% and a specificity of 88% at a cut-off score of 10 or higher^32^. The PSS-4 includes 4 items that assess the frequency of experiencing certain stressful situations over the past month using a 5-point Likert rating scale ranging from 0 to 4 for ‘never’, ‘almost never’, ‘sometimes’, ‘fairly often’, and ‘very often’^30^. The total PSS-4 score, ranging from 0 to 16, is summed by the scores of the four questions, with a higher PSS-4 score indicating a greater level of perceived stress. Although no cut-off scores were established, a PSS-4 score of 6, based on the normative score of an English population, was used to classify high levels of stress^33^. PC-PTSD includes four items asking if respondents have experienced symptoms of PTSD in the past month (yes/no)^31^. A sum score ≥ 3 (i.e., respondents answered “yes” for three or more items) was defined as positive for symptoms of trauma-related disorders, with reasonable sensitivity (78%) and specificity (89%)^31^. The Cronbach’s alphas for GAD-7, PHQ-9, PSS-4, and PC-PTSD were 0.93, 0.90, 0.77, and 0.67, respectively, indicating good internal consistency (Table S1).

### Measurement of insecurity, social support, and change in relationships

We assessed insecurity since the pandemic by asking the participants how much they worry about (1) having enough money to cover their living costs, (2) health insurance, and (3) having enough food and other grocery items. Participants had three options to choose from: not at all, a little bit, or extremely worried. A participant was classified as having any insecurity of money, health insurance, and/or food as long as they reported being a little bit or extremely worried about any of these three items.

The Oslo Social Support Scale (OSSS-3) was used to evaluate social support^34^. The OSSS-3 contains three items, covering the primary support group (How many people are you so close to that you can count on them if you have great personal problems, scoring from 1 to 4 for ‘none’, ‘1–2’, ‘3–5’, and ‘ > 5’, respectively), interest and concern shown by others (How much interest and concern do people show in what you do, scoring from 1 to 5 for ‘none’, ‘little’, ‘uncertain’, ‘some’, and ‘a lot’, respectively), and ease of obtaining practical help (How easy is it to get practical help from neighbors if you should need it, scoring from 1 to 5 for ‘very difficult’, ‘difficult’, ‘possible’, ‘easy’, ‘very easy’, respectively)^35^. The total score ranges from 3 to 14, with low scores indicating poor levels of social support. The OSSS-3 scores were split into three categories: poor (3–8), moderate (9–11), and strong social support (12–14). The Cronbach’s alphas for OSSS-3 in our study was 0.64 (Table S1).

We asked about participants’ subjective changes in relationships with parents, children, and significant others, respectively, since the beginning of the pandemic (not applicable, better, no change, worse). We further combined responses to the three questions above into a single variable with four levels: “No change” indicates no change in relationships; “Better, no worse” indicates only better and no worse relationships; “Mixed” indicates both better and worse relationships; “Worse, no better” indicates only worse and no better relationships.

### Measurement of covariates

Participants were asked for socio-demographic information, including age (continuous), sex (male, female), race/ethnicity (Non-Hispanic White, Non-Hispanic Black, Hispanic, Asian/Pacific Islander, other [including participants self-identifying as races not listed above or multi-racial]), annual household income (< $20k, $20 to < $50k, $50 to  < $75k, $75–$150k, $150 to  < $225k, ≥ $225k), education levels (high school degree or below, some college/vocational school, college graduate, graduate or higher), marital status (single, married/living together, separated/divorced/widowed), and employment status (no, yes, retired). Quarantine status at the time of completing the questionnaire was measured, and the question read, “which of the following is your current status?” The quarantine status was categorized as no restriction, stayed at home most of the time but went to work/school sometimes, stayed at home almost all the time, and isolation because of exposure history.

### Statistical analysis

We calculated means and standard deviations (SD) for each indicator of poor mental health. Pearson correlation was used to assess correlations between each pair of scales. The prevalence of symptoms of anxiety (GAD-7 ≥ 10), depression (PHQ-9 ≥ 10), stress (PSS-4 ≥ 6), and trauma-related disorders (PC-PTSD ≥ 3) were compared with chi-square tests. Logistic regression models were fit to evaluate whether insecurity of money, health insurance and/or food, social support, and change in relationships (independent variables) were associated with each indicator of poor mental health (dependent variables), adjusting for age, sex, race/ethnicity, annual household income, education level, marital status, and employment status; we also adjusted for quarantine status in the model as it was associated with symptoms of anxiety, depression, and trauma-related disorders in our previous study^26^. We analyzed the effects of change in each kind of family relationship separately. We also analyzed the change in any relationships, where the odds of poor mental health among people with only worsened, mixed (better in one or more relationships while worse in others), or only improved relationships were compared to those who did not have changes in their relationships.

Stratified analyses were conducted to examine effect modification by age (18–26, 27–64, ≥ 65 years), race/ethnicity (non-Hispanic White, non-White), and sex (male, female). We created product terms by multiplying each exposure and effect modifier of interest. Multiplicative interaction was considered present if the product terms included in the multivariate models met statistical significance.

In the sensitivity analysis, we used linear regression models to examine the associations of insecurity, social support, and change in relationships with continuous PSS-4 score, as no cut-offs for PSS-4 score have been established. In addition, because non-Hispanic Black participants presented the lowest prevalence of symptoms of anxiety, stress, and trauma-related disorders in our study, we further performed stratified analyses by non-Hispanic White, non-Hispanic Black, and other race/ethnicity participants.

Variance Inflation Factor (VIF) was used to check the multicollinearity of variables. We did not detect multicollinearity as all VIFs were below 2. Bonferroni correction was used to account for multiple comparisons. All statistical analyses were conducted using SAS 9.4 (SAS Institute Inc, Cary, NC).

## Results

A total of 3,952 participants aged 18–96 years (mean [SD]: 52.2 [16.8]) were included in our study. The geographic distribution of study participants is described in Fig. S1. The mean (SD) scores for GAD-7, PHQ-9, PSS-4, and PTSD-PC were 5.90 (5.59), 6.74 (6.16), 5.73 (3.53), and 1.34 (1.34), respectively. Scores were moderately or highly correlated (Pearson *r* = 0.46–0.78, all *P* values < 0.001) (Table S2). The prevalence of self-reported symptoms of anxiety, depression, stress, and trauma-related disorders were 22.8%, 26.7%, 51.0%, and 21.8%, respectively. The presence of poor mental health was higher among participants who were younger, female, with lower levels of education, lower household income, and never married/separated/divorced/widowed (all *P* values < 0.05) (Table 1). The prevalence of symptoms of anxiety, depression, and stress was higher among Hispanic, Asian/Pacific Islander and other race/ethnicity as compared to non-Hispanic White and non-Hispanic Black.

**Table 1:** Prevalence of symptoms of poor mental health by population characteristics.

Characteristics	Population distribution^a^	Prevalence			
		GAD-7 ≥ 10^b^	PHQ-9 ≥ 10^b^	PSS-4 ≥ 6^b^	PC-PTSD ≥ 3^b^
Overall	3952	899 (22.8)	1054 (26.7)	2017 (51.0)	862 (21.8)
Age groups					
18–26	283 (7.2)	102 (36.0)	126 (44.5)	190 (67.1)	93 (32.9)
27–44	1115 (28.2)	375 (33.6)	394 (35.3)	706 (63.3)	345 (30.9)
45–64	1449 (36.7)	318 (21.9)	379 (26.2)	716 (49.4)	301 (20.8)
≥ 65	1105 (28.0)	104 (9.4)	155 (14.0)	405 (36.7)	123 (11.1)
* P*		** < 0.0001**	** < 0.0001**	** < 0.0001**	** < 0.0001**
Sex					
Male	835 (21.1)	145 (17.4)	177 (21.2)	378 (45.3)	126 (15.1)
Female	3117 (78.9)	754 (24.2)	877 (28.1)	1639 (52.6)	736 (23.6)
* P*		** < 0.0001**	** < 0.0001**	** < 0.001**	** < 0.0001**
Race/ethnicity					
Non-Hispanic White	3240 (82.0)	714 (22.0)	835 (25.8)	1617 (49.9)	700 (21.6)
Non-Hispanic Black	227 (5.7)	42 (18.5)	59 (26.0)	104 (45.8)	47 (20.7)
Hispanic	101 (2.6)	28 (27.2)	36 (35.6)	65 (64.4)	32 (31.7)
Asian/Pacific Islander	106 (2.7)	27 (25.5)	33 (31.1)	67 (63.2)	24 (22.6)
Other^c^	243 (6.2)	75 (30.9)	80 (32.9)	138 (56.8)	54 (22.2)
* P*		**0.01**	**0.02**	** < 0.001**	0.19
Education					
High school degree or below	144 (3.6)	59 (41.0)	63 (43.8)	90 (62.5)	46 (31.9)
Some college/vocational school	650 (16.5)	194 (29.8)	238 (36.6)	396 (60.9)	154 (23.7)
College graduate	1434 (36.3)	317 (22.1)	367 (25.6)	732 (51.0)	325 (22.7)
Graduate or higher	1724 (43.6)	329 (19.1)	386 (22.4)	799 (46.3)	337 (19.5)
* P*		** < 0.0001**	** < 0.0001**	** < 0.0001**	**0.001**
Annual household income					
Less than $20K	280 (7.1)	110 (39.3)	147 (52.5)	200 (71.4)	95 (33.9)
$20K to $49.9K	763 (19.3)	211 (27.7)	260 (34.1)	442 (57.9)	182 (23.9)
$50K to $74.9K	817 (20.7)	190 (23.3)	219 (26.8)	415 (50.8)	170 (20.8)
$75K to $149.9K	1320 (33.4)	266 (20.2)	306 (23.2)	648 (49.1)	280 (21.2)
$150K to $224.9K	473 (12.0)	81 (17.1)	76 (16.1)	203 (42.9)	80 (16.9)
$225K and over	219 (5.5)	32 (14.6)	34 (15.5)	84 (38.4)	44 (20.1)
* P*		** < 0.0001**	** < 0.0001**	** < 0.0001**	** < 0.0001**
Marital status					
Single	932 (23.6)	292 (31.3)	366 (39.3)	605 (64.9)	276 (29.6)
Married/living together	2166 (54.8)	445 (20.5)	457 (21.1)	1008 (46.5)	429 (19.8)
Separated/divorced/widowed	848 (21.5)	160 (18.9)	229 (27.0)	402 (47.4)	155 (18.3)
* P*		** < 0.0001**	** < 0.0001**	** < 0.0001**	** < 0.0001**
Employment status					
No	397 (10.1)	141 (35.5)	156 (39.3)	260 (65.5)	123 (31.0)
Yes	2227 (56.4)	524 (23.5)	580 (26.0)	1159 (52.0)	502 (22.5)
Retired	1317 (33.3)	234 (17.8)	315 (23.9)	592 (45.0)	236 (17.9)
* P*		** < 0.0001**	** < 0.0001**	** < 0.0001**	** < 0.0001**

*GAD-7* Generalized Anxiety Disorder Scale-7 items, *PHQ-9* Patient Health Questionnaire-9 items, *PSS-4* Perceived Stress Scale 4, *PC-PTSD* primary care PTSD screen, *PTSD* post-traumatic stress disorder.

^a^Percentages may not add up to 100% owing to missing values (n = 35 in race/ethnicity, n = 80 in annual household income, n = 6 in marital status, n = 11 in employment status).

^b^Percentages represent the prevalence.

^c^Participants self-identified as races not listed above or multi-racial.

Significant values are in bold.

More than half of participants (57.9%) reported insecurity (a little bit or extremely worried about money, health insurance, and/or food), and 10.1%, 7.6%, and 5.4% reported being extremely worried about money, health insurance, or food, respectively (Table 2). Insecurity of money, health insurance, and/or food was associated with 3.74 (95% CI: 3.06, 4.56), 3.20 (95% CI: 2.67, 3.84), 3.08 (95% CI: 2.67, 3.57), and 2.93 (95% CI: 2.42, 3.55) times the odds of symptoms of anxiety, depression, stress, and trauma-related disorders (Table 2). Participants who were a little bit worried or extremely worried about money, health insurance, or food had higher odds of all symptoms than those who were not worried at all.

**Table 2:** Odds ratios (ORs) and confidence intervals (CIs) of poor mental health in association with insecurity, social support, and change in relationships.

Characteristics	Population distribution	GAD-7 ≥ 10	PHQ-9 ≥ 10	PSS-4 ≥ 6	PC-PTSD ≥ 3
Any insecurity^a^					
No	1661 (42.1)	Ref	Ref	Ref	Ref
Yes	2283 (57.9)	**3.74 (3.06, 4.56)**^**#**^	**3.20 (2.67, 3.84)**^**#**^	**3.08 (2.67, 3.57)**^**#**^	**2.93 (2.42, 3.55)**^**#**^
Worry about money^a,b^					
Not worried at all	2112 (53.6)	Ref	Ref	Ref	Ref
A little bit worried	1431 (36.2)	**1.94 (1.57, 2.41)**^**#**^	**2.00 (1.64, 2.44)**^**#**^	**2.07 (1.75, 2.46)**^**#**^	**1.53 (1.24, 1.88)**^**#**^
Extremely worried	397 (10.1)	**4.48 (3.23, 6.22)**^**#**^	**3.86 (2.80, 5.32)**^**#**^	**4.48 (3.09, 6.49)**^**#**^	**2.29 (1.65, 3.18)**^**#**^
* P* for trend		** < 0.0001**	** < 0.0001**	** < 0.0001**	** < 0.0001**
Worry about health insurance^a,c^					
Not worried at all	2706 (68.6)	Ref	Ref	Ref	Ref
A little bit worried	940 (23.8)	**1.31 (1.07, 1.58)**	**1.30 (1.07, 1.58)**	**1.32 (1.10, 1.59)**	**1.38 (1.13, 1.70)**
Extremely worried	299 (7.6)	**1.76 (1.27, 2.43)**^**#**^	**1.47 (1.07, 2.03)**	**2.51 (1.69, 3.72)**^**#**^	**2.15 (1.57, 2.95)**^**#**^
* P* for trend		**0.0002**	**0.0022**	** < 0.0001**	** < 0.0001**
**Worry about food**^**a,d**^					
Not worried at all	2621 (66.4)	Ref	Ref	Ref	Ref
A little bit worried	1113 (28.2)	**1.55 (1.27, 1.89)**^**#**^	**1.49 (1.23, 1.80)**^**#**^	**1.44 (1.21, 1.71)**^**#**^	**1.64 (1.34, 2.00)**^**#**^
Extremely worried	215 (5.4)	**2.79 (1.89, 4.11)**^**#**^	**2.56 (1.74, 3.77)**^**#**^	**3.84 (2.21, 6.66)**^**#**^	**2.53 (1.73, 3.69)**^**#**^
* P* for trend		** < 0.0001**	** < 0.0001**	** < 0.0001**	** < 0.0001**
Social support^a^					
Poor	1155 (29.3)	Ref	Ref	Ref	Ref
Moderate	1797 (45.6)	**0.39 (0.32, 0.47)**^**#**^	**0.33 (0.27, 0.39)**^**#**^	**0.40 (0.34, 0.47)**^**#**^	**0.64 (0.53, 0.77)**^**#**^
Strong	985 (25.0)	**0.32 (0.25, 0.40)**^**#**^	**0.20 (0.16, 0.26)**^**#**^	**0.23 (0.18, 0.28)**^**#**^	**0.57 (0.46, 0.72)**^**#**^
* P* for trend		** < 0.0001**	** < 0.0001**	** < 0.0001**	** < 0.0001**
Change in relationship with parents ^a,e^^,^*					
No change	1619 (67.8)	Ref	Ref	Ref	Ref
Better	437 (18.3)	1.20 (0.92, 1.57)	1.05 (0.81, 1.37)	1.17 (0.92, 1.50)	1.14 (0.87, 1.48)
Worse	331 (13.9)	**2.43 (1.85, 3.21)**^**#**^	**1.66 (1.25, 2.19)**^**#**^	**1.59 (1.19, 2.11)**	**2.25 (1.71, 2.94)**^**#**^
Change in relationship with children^a,f^^,^*					
No change	1369 (61.0)	Ref	Ref	Ref	Ref
Better	627 (28.0)	1.27 (0.94, 1.72)	1.13 (0.84, 1.51)	1.26 (1.00, 1.60)	1.29 (0.96, 1.73)
Worse	247 (11.0)	**2.55 (1.78, 3.66)**^**#**^	**2.59 (1.82, 3.68)**^**#**^	**2.21 (1.58, 3.10)**^**#**^	**1.87 (1.30, 2.69)**
Change in relationship with significant others ^a,g^^,^*					
No change	1400 (52.5)	Ref	Ref	Ref	Ref
Better	830 (31.1)	1.11 (0.86, 1.43)	0.98 (0.76, 1.27)	1.02 (0.83, 1.25)	1.2 (0.93, 1.53)
Worse	438 (16.4)	**2.85 (2.16, 3.77)**^**#**^	**3.05 (2.31, 4.02)**^**#**^	**3.14 (2.39, 4.12)**^**#**^	**1.76 (1.32, 2.33)**^**#**^
Change in relationship^a,h^					
No change	1686 (46.0)	Ref	Ref	Ref	Ref
Better, no worse	1159 (31.6)	**1.36 (1.10, 1.68)**	1.13 (0.92, 1.37)	1.18 (1.00, 1.39)	**1.33 (1.08, 1.60)**
Mixed	243 (6.6)	**3.44 (2.50, 4.71)**^**#**^	**2.12 (1.55, 2.92)**^**#**^	**2.67 (1.97, 3.63)**^**#**^	**2.40 (1.72, 3.23)**^**#**^
Worse, no better	576 (15.7)	**3.91 (3.11, 4.92)**^**#**^	**3.14 (2.51, 3.92)**^**#**^	**2.94 (2.37, 3.66)**^**#**^	**2.23 (1.77, 2.80)**^**#**^

Bold indicates *P* < 0.05 prior to Bonferroni correction.

*GAD-7* Generalized Anxiety Disorder Scale-7 items, *PHQ-9* Patient Health Questionnaire-9 items, *PSS-4* Perceived Stress Scale 4, *PC-PTSD* primary care PTSD screen, *PTSD* post-traumatic stress disorder.

*Participants who choose “Not applicable” to questions on change in family relationships were excluded from analyses.

^#^*P* < 0.05 after Bonferroni correction.

^a^Adjusted for age, sex, race/ethnicity, education, annual household income, marital status, employment status, and quarantine status.

^b^Adjusted for worry about health insurance and food.

^c^Adjusted for worry about money and food.

^d^Adjusted for worry about money and health insurance.

^e^Adjusted for change in relationship with children and significant others.

^f^Adjusted for change in relationship with parents and significant others.

^g^Adjusted for change in relationship with parents and children.

^h^“No change” indicates participants did not change relationships with parents, children, or significant others. “Better, no worse” indicates participants had better and no worse relationships with parents, children, and significant others. “Mixed” indicates participants had both better and worse relationships with parents, children, and significant others. “Worse, no better” indicates participants had worse and no better relationships with parents, children, and significant others.

Compared to those with poor social support, participants with strong and moderate social support experienced lower odds of symptoms of anxiety, depression, stress, and trauma-related disorders (Table 2). For example, moderate (OR = 0.40, 95% CI: 0.34, 0.47) and strong (OR = 0.23, 95% CI: 0.18, 0.28) social support were associated with lower odds of symptoms of stress, respectively. The results were comparable in the sensitivity analysis when continuous PSS-4 scores were used as the response variable (Table S3).

Participants whose relationships with parents, children, or significant others became worse during the pandemic experienced increased odds of poor mental health (Table 2). We further examined the association between any change in relationships with parents, children, or significant others (better, mixed, or worse) and poor mental health. Compared to those without any change in relationships, those who experienced only worse relationships (worse and no better relationships with parents, children, and/or significant others) or mixed relationships (both better and worse relationships with parents, children, and/or significant others) had higher odds of poor mental health. Those who reported better and no worse relationships with parents, children, and/or significant others also had higher odds of symptoms of anxiety (OR = 1.36; 95% CI: 1.10, 1.68) and trauma-related disorders (OR = 1.33; 95% CI: 1.08, 1.60), but these associations were not statistically significant after adjusting for multiple comparisons.

**Figure 1:**
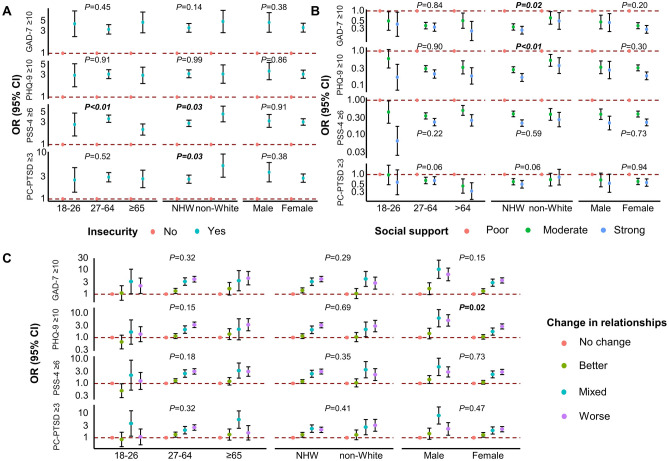
Associations of (**A**) insecurity, (**B**) social support, and (**C**) change in relationships with indicators of poor mental health stratified by age groups, racial/ethnic groups, and sex. Dots represent estimated ORs for symptoms of anxiety (GAD-7 ≥ 10), depression (PHQ-9 ≥ 10), stress (PSS-4 ≥ 6), and trauma-related disorders (PC-PTSD score ≥ 3), and error bars construct 95% CIs. Dashed lines indicate the null association (OR = 1). *GAD-7* Generalized Anxiety Disorder Scale-7 items, *PHQ-9* Patient Health Questionnaire-9 items, *PSS-4* Perceived Stress Scale 4, *PC-PTSD* primary care PTSD screen, *PTSD* post-traumatic stress disorder, *OR* odds ratio, *CI* confidence interval, *NHW* non-Hispanic White. *P* values represent crude *P* for interaction terms. None of *P* values for interaction was statistically significant after Bonferroni correction.

In the stratified analysis (Fig. 1 and Table S4), the associations of insecurity of money, health insurance, and/or food and symptoms of stress were stronger among people aged < 65 years compared to people aged 65 years and over (crude *P* for interaction < 0.01). Stronger associations of insecurity with symptoms of stress or trauma-related disorders were found among non-White than White participants (both crude *P* for interaction = 0.03). We also found that the associations between strong social support and lower odds of symptoms of anxiety and depression were more pronounced among White participants than non-Whites (crude *P* values for interaction were 0.02 and < 0.01 for symptoms of anxiety and depression, respectively). The association between change in any relationships and depressive symptoms was stronger among males than females (crude* P* for interaction = 0.02). However, none of the *P* values for interaction terms was statistically significant after adjusting for multiple comparisons (all Bonferroni-adjusted *P* values > 0.05). In the sensitivity analysis, non-Hispanic Black participants appeared to be more affected by insecurity and worse family relationships than White or other racial/ethnic participants, but the confidence intervals were very wide due to the small sample size, making it difficult to draw a conclusion among specific race/ethnicity groups (Fig. S2).

## Discussion

In this study, we found disparities in COVID-19-related poor mental health, with a higher prevalence among those who were younger, female, with lower socioeconomic status, and racial/ethnic minorities. We also found that insecurity of money, health insurance, and/or food and changes in family relationships were associated with poor mental health during the pandemic, while social support presented as a protective factor for poor mental health. These associations may differ by age, race/ethnicity, and sex.

The COVID-19 pandemic has had significant consequences on mental health. For example, the prevalence of depressive symptoms was threefold higher in April 2020 (27.8%) compared with that before the COVID-19 pandemic (8.5%)^6^. Our online survey conducted between May and August 2020 also observed comparable results (the prevalence of depressive symptoms: 26.7%). Factors such as social isolation, economic instability, disruption of daily routines, and the limited availability of vaccines have contributed to poor mental health in the early phase of the pandemic. Our results align with previous studies that observed substantial disparities in poor mental health during the pandemic^36–38^. Due to lockdowns, young people have faced significant disruptions to their education, social lives, and daily routines, which can contribute to poor mental health^39^. Low levels of household income were found to be associated with poor mental health in previous research^40^. During the pandemic, lower-income individuals have experienced higher rates of job loss, food insecurity, and housing instability, further exacerbating poor mental health, such as symptoms of anxiety and depression^6,41^. A number of studies have reported the disproportionate impact of COVID-19 on poor mental health among racial and ethnic minorities^42^. Long-standing structural racism and pre-existing health disparities (e.g., lower access to healthcare, higher uninsured rates), coupled with greater exposure to pandemic-related stressors (e.g., unemployment, food insecurity, discrimination) have predisposed racial and ethnic minorities to worse mental health^43,44^. Consistent with other studies, a higher prevalence of poor mental health were found among Hispanic and Asian compared to non-Hispanic White^6,23,45,46^. We found that non-Hispanic Black participants reported fewer or comparable levels of symptoms to White participants, which aligns with findings from both pre-pandemic and COVID-19-related research^47,48^. Despite experiencing greater pandemic-related stressors, poor mental health was not more prevalent among Black respondents^47^. This phenomenon could be attributed to the African American community’s higher levels of resilience and stronger religious support^48^. In a COVID-19-related study, Black respondents had significantly greater odds of high resilience levels compared to White respondents, which were, in turn, associated with less mental distress^49^.

Since the pandemic, there have been increasing concerns about mental health resulting from food insecurity, financial uncertainty, and health insurance insecurity^1–4^. Consistent with those studies, in our sample, about 28% of participants reported being a little bit worried about food, and 5% were extremely worried. In line with previous research, food insecurity was associated with higher odds of symptoms of anxiety and depression^9^. In addition, given the high unemployment rates during the pandemic, people may also experience financial concerns and health insurance coverage losses. In a previous survey in April 2020, 32% of employed individuals reported having some degree of concern about finances over the next 12 months^4^. About 46% and 33% of participants in our study were worried about money and health insurance to some extent, respectively. Greater concern regarding their financial situation and health insurance coverage was associated with an increased risk of poor mental health after adjusting for household income and employment status, which was consistent with other studies^4^. We found that non-White participants were more susceptible to the adverse effects of insecurity about money, health insurance, and food on symptoms of stress and trauma-related disorders, and this may be because relative to White individuals, racial/ethnic minorities were at a higher risk of food insecurity, insufficient money, and being underinsured or uninsured^50–52^, which could result in or worsen poor mental health^4,53,54^.

Perceived social support has been considered protective against poor mental health^16,55,56^. Social support might enhance resilience to stress trauma and buffer poor mental health even under stressful physical and psychosocial circumstances^57,58^. Our findings of moderate or strong social support in association with decreased odds of poor mental health highlighted the importance of social support systems in relieving poor mental health and coping with the pandemic. In addition, our study suggested that high levels of social support for non-White individuals may have a less protective effect on poor mental health than in White individuals. Racial/ethnic minorities tend to seek support from communities and are less likely than White individuals to have a robust social support system^59^. Compared to White individuals, racial/ethnic minorities may have received lower levels of social support amid the pandemic due to restricted in-person meetings following stay-at-home orders. However, our findings may be due to chance since interaction terms were not significant after adjusting for multiple comparisons. The racial/ethnic differences in the associations between social support and mental health remain inconclusive and merit further investigation.

We found that changes in relationships with parents, children, or significant others were associated with poor mental health. Staying at home may result in more spare time spent with family members and improvement of family relationships, including family (re)connection and acknowledgment, better communication, and emotional expressiveness^60–62^. Good quality of family relationships, such as marital satisfaction and satisfying relationships with adult children, contribute to psychological well-being^63^. In previous studies, positive family relationships could potentially protect against mental distress in the context of the pandemic^64–66^. In our study, participants experiencing worse relationships presented poorer mental health than those with better or no changes in family relationships. Targeted intervention strategies are needed to promote the quality of family relationships and manage COVID-19-related psychological problems. However, better and no worse relationships with parents, children, and/or significant others were also associated with higher odds of poor mental health, and this was probably because we were unable to account for other unmeasured factors. For example, due to lockdown measures, people had to meet various demands simultaneously (e.g., parenting, working, and studying remotely) and potentially had to compromise time for self-care^67,68^, which may contribute to poor mental health. Another potential explanation may be that individuals who were suffering psychological distress may have been more motivated to seek social support and engage with their social network, and consequently, the relationship quality improved. Furthermore, our analysis was only focused on change in relationships without controlling for relationship status prior to the pandemic. Participants whose family relationships improved from poor quality might still face worse mental health compared to those who had neutral or positive family relationships before the pandemic and experienced further improvements during the pandemic. Besides, we only measured subjective changes, and participants' perceptions of relationship changes may differ. Chance findings cannot be ruled out either since these associations were no longer significant adjusting for multiple testing. Future studies that examine relationship changes in terms of quantity (i.e., losing a family member or divorce) may provide more comprehensive insights into the impact of family relationship changes on mental health during the pandemic.

Study limitations have to be acknowledged. First, this was a cross-sectional study and we were unable to determine the temporality in the observed associations. Second, our convenience sample consisted of individuals with access to our online survey, making it difficult to determine the response rate. This may have led to a self-selection bias, as participants were likely to have poorer mental health, higher levels of insecurity, poorer social support, and worse family relationships. Third, indicators of poor mental health were self-reported and could introduce measurement errors. Unlike mental disorders, which are more persistent and severe, indicators of poor mental health measured in our study may be temporary and may not represent participants’ long-term poor mental health. Fourth, residual and unmeasured confounding may exist in the observed associations. Fifth, our study population was predominantly comprised of White females with high education levels, and study findings may not be generalized to other populations. Lastly, due to the small sample sizes of non-White participants, we were unable to explore associations by specific race/ethnic groups. The prevalence of poor mental health was not homogeneous among non-White individuals, as some racial/ethnic groups may have experienced poorer mental health during the pandemic. Future research that includes more diverse racial and ethnic disadvantaged groups may further enhance our understanding of disparities in COVID-19-related poor mental health.

## Conclusions

Our study found a higher prevalence of poor mental health among younger individuals, females, those of lower socioeconomic status, and racial/ethnic minorities. Insecurity (concerns about money, health insurance, and/or food) and worse family relationships may have contributed to poor mental health, while social support appeared to help mitigate the negative effects on mental health during the pandemic. Targeted interventions aimed at reducing insecurity of money, health insurance, and food, enhancing social support, and improving family relationships could help alleviate poor mental health during the pandemic.

## Supplementary Information

Supplementary Information.

## Data Availability

Data are available from the corresponding author on reasonable request.
