# Supplementary material for: Disparities in insecurity, social support, and family relationships in association with poor mental health among US adults during the COVID-19 pandemic
Source: Sci Rep. 2023 Jun 15;13:9731. doi: 10.1038/s41598-023-35981-0 (PMC10272217; doi:10.1038/s41598-023-35981-0)
Supplement: Supplementary file 1 — Supplementary Information. [file 41598_2023_35981_MOESM1_ESM.docx]

**Table S1. Items wording and descriptive statistics, and internal consistency** **of instruments used in the current study**

| **Item** | **Mean** | **SD** | **Corrected item total correlation** | **Cronbach Alpha if item deleted** | **Cronbach Alpha** |
| --- | --- | --- | --- | --- | --- |
| **Generalized Anxiety Disorder 7-item (GAD-7)** |  |  |  |  |  |
| 1. Feeling nervous, anxious, or on edge | 1.09 | 1.01 | 0.88 | 0.91 |  |
| 2. Not being able to stop or control worrying | 0.73 | 0.95 | 0.89 | 0.91 |  |
| 3. Worrying too much about different things | 0.91 | 0.98 | 0.89 | 0.91 |  |
| 4. Trouble relaxing | 0.97 | 1.01 | 0.86 | 0.91 |  |
| 5. Being so restless that it's hard to sit still | 0.55 | 0.87 | 0.73 | 0.93 |  |
| 6. Becoming easily annoyed or irritable | 0.97 | 0.96 | 0.76 | 0.92 |  |
| 7. Feeling afraid as if something awful might happen | 0.69 | 0.92 | 0.81 | 0.92 |  |
| *Total GAD-7 score* | *5.90* | *5.59* |  |  | *0.93* |
| **Patient Health Questionnaire-9 (PHQ-9)** |  |  |  |  |  |
| 1. Little interest or pleasure in doing things | 0.80 | 0.94 | 0.82 | 0.88 |  |
| 2. Feeling down, depressed, or hopeless | 0.83 | 0.95 | 0.83 | 0.88 |  |
| 3. Trouble falling or staying asleep, or sleeping too much | 1.15 | 1.10 | 0.74 | 0.89 |  |
| 4. Feeling tired or having little energy | 1.22 | 1.06 | 0.79 | 0.88 |  |
| 5. Poor appetite or overeating | 0.95 | 1.07 | 0.74 | 0.89 |  |
| 6. Feeling bad about yourself – or that you are a failure | 0.63 | 0.95 | 0.77 | 0.88 |  |
| 7. Trouble concentrating on things | 0.77 | 0.96 | 0.78 | 0.88 |  |
| 8. Moving or speaking so slowly that other people could have noticed | 0.21 | 0.59 | 0.60 | 0.90 |  |
| 9. Thoughts that you would be better off dead or of hurting yourself | 0.18 | 0.56 | 0.57 | 0.90 |  |
| *Total PHQ-9 score* | *6.74* | *6.16* |  |  | *0.90* |
| **Perceived Stress Scale 4 (PSS-4)** |  |  |  |  |  |
| 1. That you were unable to control the important things in your life | 1.48 | 1.26 | 0.80 | 0.70 |  |
| 2. Confident about your ability to handle your personal problems (reverse scored) | 1.36 | 1.16 | 0.72 | 0.74 |  |
| 3. Things were going your way (reverse scored) | 1.80 | 1.01 | 0.76 | 0.70 |  |
| 4. Difficulties were piling up so high that you could not overcome them | 1.09 | 1.16 | 0.78 | 0.71 |  |
| *Total PSS-4 score* | *5.73* | *3.53* |  |  | *0.77* |
| **Primary Care Post-Traumatic Stress Disorder Screen (PC-PTSD)** |  |  |  |  |  |
| 1. Have had nightmares about the pandemic or thought about the pandemic when you did not want to | 0.28 | 0.45 | 0.69 | 0.61 |  |
| 2. Tried hard not to think about the pandemic or went out of your way to avoid situations that reminded you of the pandemic | 0.39 | 0.49 | 0.73 | 0.60 |  |
| 3. Been constantly on guard, watchful, or easily startled | 0.30 | 0.46 | 0.73 | 0.59 |  |
| 4. Felt numb or detached from others, activities, or your surroundings | 0.37 | 0.48 | 0.70 | 0.63 |  |
| *Total PTSD-PC score* | *1.34* | *1.34* |  |  | *0.67* |
| **Oslo Social Support Scale (OSSS-3)** |  |  |  |  |  |
| 1. How many people are you so close to that you can count on them if you have great personal problems? | 2.81 | 0.80 | 0.73 | 0.48 |  |
| 2. How much interest and concern do people show in what you do? | 3.60 | 1.13 | 0.79 | 0.51 |  |
| 3. How easy is it to get practical help from neighbors if you should need it? | 3.30 | 1.18 | 0.76 | 0.64 |  |
| *Total OSSS-3 score* | *9.71* | *2.38* |  |  | *0.64* |

**Table S2. Descriptive statistics and correlations of instruments**

|  | **Descriptive statistics** | | | **Pearson correlation** | | | |
| --- | --- | --- | --- | --- | --- | --- | --- |
|  | Mean | SD | Median | GAD-7 | PHQ-9 | PSS-4 | PTSD-PC |
| GAD-7 | 5.90 | 5.59 | 4 | 1 |  |  |  |
| PHQ-9 | 6.74 | 6.16 | 5 | 0.78^*^ | 1 |  |  |
| PSS-4 | 5.73 | 3.53 | 6 | 0.66^*^ | 0.69^*^ | 1 |  |
| PTSD-PC | 1.34 | 1.34 | 1 | 0.60^*^ | 0.53^*^ | 0.46^*^ | 1 |

Abbreviations: GAD-7, Generalized Anxiety Disorder 7-item; PHQ-9: Patient Health Questionnaire-9; PSS-4: Perceived Stress Scale 4; PC-PTSD: Primary Care Post-Traumatic Stress Disorder Screen.

^*^*P* <0.001

**Table S3. Association of with insecurity, social support, and change in relationships with scores of Perceived Stress Scale-4 (PSS-4)**

| **Characteristics** | **Mean (SD) of PSS-4 score** | **Beta (95% CI)** |
| --- | --- | --- |
| **Any insecurity ^a^** |  |  |
| No | 4.18 (3.06) | ref |
| Yes | 6.86 (3.41) | **2.26 (2.04, 2.47) ^#^** |
| **Worry about money ^a,b^** |  |  |
| Not worried at all | 4.47 (3.14) | ref |
| A little bit worried | 6.69 (3.26) | **1.36 (1.11, 1.61) ^#^** |
| Extremely worried | 8.96 (3.22) | **2.48 (2.04, 2.92) ^#^** |
| *P* for trend |  | **<.0001** |
| **Worry about health insurance ^a,c^** |  |  |
| Not worried at all | 5.01 (3.32) | ref |
| A little bit worried | 6.82 (3.33) | **0.61 (0.36, 0.87) ^#^** |
| Extremely worried | 8.82 (3.43) | **1.27 (0.83, 1.71) ^#^** |
| *P* for trend |  | **<.0001** |
| **Worry about food ^a,d^** |  |  |
| Not worried at all | 4.93 (3.3) | ref |
| A little bit worried | 6.90 (3.34) | **0.67 (0.42, 0.92) ^#^** |
| Extremely worried | 9.40 (3.08) | **1.66 (1.13, 2.19) ^#^** |
| *P* for trend |  | **<.0001** |
| **Social support ^a^** |  |  |
| Poor | 7.51 (3.63) | ref |
| Moderate | 5.39 (3.19) | **-1.91 (-2.15, -1.17) ^#^** |
| Strong | 4.25 (3.11) | **-2.95 (-3.23, -2.67) ^#^** |
| *P* for trend |  | **<.0001** |
| **Change in relationship with parents ^a,e^**^*^ |  |  |
| No change | 5.76 (3.42) | ref |
| Better | 6.39 (3.42) | 0.23 (-0.14, 0.60) |
| Worse | 7.92 (3.88) | **1.30 (0.89, 1.70) ^#^** |
| **Change in relationship with children ^a,f^**^*^ |  |  |
| No change | 4.81 (3.35) | ref |
| Better | 5.31 (3.13) | 0.23 (-0.11, 0.58) |
| Worse | 7.39 (3.54) | **1.40 (0.93, 1.87) ^#^** |
| **Change in relationship with significant others ^a,g^**^*^ |  |  |
| No change | 4.89 (3.21) | ref |
| Better | 5.48 (3.29) | 0.05 (-0.25, 0.35) |
| Worse | 7.91 (3.46) | **2.20 (1.84, 2.57) ^#^** |
| **Change in relationship ^a,h^** |  |  |
| No change | 4.98 (3.31) | ref |
| Better, no worse | 5.51 (3.26) | **0.33 (0.08, 0.58)** |
| Mixed | 6.88 (3.28) | **1.67 (1.22, 2.11)** **^#^** |
| Worse, no better | 7.85 (3.67) | **2.42 (2.11, 2.73) ^#^** |

^a^ Adjusted for age, sex, race/ethnicity, education, annual household income, marital status, employment status, and quarantine status.

^b^ Adjusted for worry about health insurance and food.

^c^ Adjusted for worry about money and food.

^d^ Adjusted for worry about money and health insurance.

^e^ Adjusted for change in relationship with children and significant others.

^f^ Adjusted for change in relationship with parents and significant others.

^g^ Adjusted for change in relationship with parents and children.

^h^ “No change” indicates participants did not change relationships with parents, children, or significant others. “Better, no worse” indicates participants had better and no worse relationships with parents, children, and significant others. “Mixed” indicates participants had both better and worse relationships with parents, children, and significant others. “Worse, no better” indicates participants had worse and no better relationships with parents, children, and significant others.

^*^ Participants who choose “Not applicable” to questions on change in family relationships were excluded from analyses.

Bold indicates *P* <0.05 prior to Bonferroni correction. ^#^ indicates *P* <0.05 after Bonferroni correction.

**Table S4. Odds ratios (ORs) and confidence intervals (CIs) of poor mental health in association with insecurity, social support, and change in relationship in subgroups ^a^**

| **Subgroup** |  | **Exposure** | **GAD-7 ≥10** | **PHQ-9 ≥10** | **PSS-4 ≥6** | **PC-PTSD ≥3** |
| --- | --- | --- | --- | --- | --- | --- |
|  |  | **Insecurity in money, health insurance, food** | | | | |
| **Age group** | 18-26 years | No | ref | ref | ref | ref |
|  |  | Yes | **4.35 (2.29, 8.24) ^#^** | **2.97 (1.67, 5.28) ^#^** | **2.71 (1.53, 4.81)** | **2.58 (1.40, 4.76)** |
|  | 27-64 years | No | ref | ref | ref | ref |
|  |  | Yes | **3.29 (2.60, 4.16) ^#^** | **3.13 (2.51, 3.92) ^#^** | **3.61 (3.01, 4.34) ^#^** | **2.96 (2.34, 3.74) ^#^** |
|  | ≥65 years | No | ref | ref | ref | ref |
|  |  | Yes | **4.66 (2.78, 7.83) ^#^** | **2.96 (1.97, 4.45) ^#^** | **2.12 (1.60, 2.79) ^#^** | **2.73 (1.76, 4.22) ^#^** |
|  | *P interaction* |  | *0.45* | *0.91* | ***0.002*** | *0.52* |
| **Race/ethnicity** | NHW | No | ref | ref | ref | ref |
|  |  | Yes | **3.55 (2.86, 4.40) ^#^** | **3.20 (2.62, 3.90) ^#^** | **2.88 (2.46, 3.37) ^#^** | **2.68 (2.18, 3.29) ^#^** |
|  | Non-White | No | ref | ref | ref | ref |
|  |  | Yes | **4.87 (2.79, 8.50) ^#^** | **3.16 (2.00, 4.98) ^#^** | **4.59 (3.11, 6.79) ^#^** | **5.26 (2.95, 9.38) ^#^** |
|  | *P interaction* |  | *0.14* | *0.99* | ***0.03*** | ***0.03*** |
| **Sex** | Male | No | ref | ref | ref | ref |
|  |  | Yes | **4.65 (2.85, 7.59) ^#^** | **3.65 (2.38, 5.61) ^#^** | **3.24 (2.35, 4.49) ^#^** | **3.79 (2.34, 6.15) ^#^** |
|  | Female | No | ref | ref | ref | ref |
|  |  | Yes | **3.59 (2.88, 4.47) ^#^** | **3.13 (2.56, 3.83) ^#^** | **3.08 (2.62, 3.63) ^#^** | **2.82 (2.29, 3.48) ^#^** |
|  | *P interaction* |  | *0.38* | *0.86* | *0.91* | *0.38* |
|  |  | **Social support** |  |  |  |  |
| **Age group** | 18-26 years | Strong | ref | ref | ref | ref |
|  |  | Moderate | **0.51 (0.28, 0.95)** | 0.60 (0.32, 1.11) | **0.45 (0.21, 0.96)** | 0.97 (0.51, 1.86) |
|  |  | Poor | **0.41 (0.18, 0.93)** | **0.17 (0.07, 0.41) ^#^** | **0.07 (0.02, 0.17) ^#^** | 0.59 (0.26, 1.36) |
|  | 27-64 years | Strong | ref | ref | ref | ref |
|  |  | Moderate | **0.37 (0.30, 0.46) ^#^** | **0.31 (0.25, 0.38) ^#^** | **0.35 (0.29, 0.43) ^#^** | **0.67 (0.54, 0.83) ^#^** |
|  |  | Poor | **0.34 (0.26, 0.44) ^#^** | **0.22 (0.16, 0.28) ^#^** | **0.23 (0.18, 0.30) ^#^** | **0.66 (0.51, 0.87)** |
|  | ≥65 years | Strong | ref | ref | ref | ref |
|  |  | Moderate | **0.52 (0.32, 0.87)** | **0.33 (0.21, 0.52) ^#^** | **0.51 (0.36, 0.71) ^#^** | **0.46 (0.29, 0.75)** |
|  |  | Poor | **0.26 (0.14, 0.50) ^#^** | **0.19 (0.11, 0.33) ^#^** | **0.26 (0.18, 0.38) ^#^** | **0.33 (0.19, 0.57) ^#^** |
|  | *P interaction* |  | *0.84* | *0.90* | *0.22* | *0.06* |
| **Race/ethnicity** | NHW | Strong | ref | ref | ref | ref |
|  |  | Moderate | **0.34 (0.28, 0.42) ^#^** | **0.29 (0.23, 0.35) ^#^** | **0.40 (0.33, 0.48) ^#^** | **0.61 (0.50, 0.76) ^#^** |
|  |  | Poor | **0.28 (0.21, 0.36) ^#^** | **0.17 (0.13, 0.22) ^#^** | **0.22 (0.17, 0.27) ^#^** | **0.52 (0.40, 0.67) ^#^** |
|  | Non-White | Strong | ref | ref | ref | ref |
|  |  | Moderate | **0.63 (0.41, 0.96)** | **0.53 (0.36, 0.79)** | **0.39 (0.26, 0.58) ^#^** | 0.71 (0.46, 1.09) |
|  |  | Poor | **0.51 (0.29, 0.90)** | **0.38 (0.22, 0.64)** | **0.27 (0.17, 0.44) ^#^** | 0.83 (0.49, 1.41) |
|  | *P interaction* |  | ***0.02*** | ***0.002*** | *0.59* | *0.06* |
| **Sex** | Male | Strong | ref | ref | ref | ref |
|  |  | Moderate | **0.48 (0.30, 0.76)** | **0.34 (0.22, 0.52) ^#^** | **0.39 (0.28, 0.56) ^#^** | 0.69 (0.44, 1.10) |
|  |  | Poor | **0.46 (0.25, 0.84)** | **0.28 (0.16, 0.50) ^#^** | **0.22 (0.14, 0.35) ^#^** | 0.56 (0.30, 1.02) |
|  | Female | Strong | ref | ref | ref | ref |
|  |  | Moderate | **0.37 (0.30, 0.46) ^#^** | **0.32 (0.27, 0.39) ^#^** | **0.40 (0.33, 0.48) ^#^** | **0.63 (0.51, 0.77) ^#^** |
|  |  | Poor | **0.29 (0.23, 0.38) ^#^** | **0.19 (0.15, 0.25) ^#^** | **0.23 (0.18, 0.28) ^#^** | **0.58 (0.45, 0.74) ^#^** |
|  | *P interaction* |  | *0.20* | *0.30* | *0.73* | *0.94* |
|  |  | **Change in relationships with parents, children, or significant others** | | | | |
| **Age group** | 18-26 years | No change | ref | ref | ref | ref |
|  |  | Better, no worse | 1.11 (0.56, 2.22) | 0.66 (0.34, 1.26) | 0.5 (0.26, 0.97) | 0.85 (0.43, 1.69) |
|  |  | Mixed | **3.31 (1.03, 10.65)** | 1.68 (0.53, 5.35) | 2.19 (0.53, 9.08) | **3.88 (1.16, 12.93)** |
|  |  | Worse, no better | **2.21 (1.06, 4.57)** | 1.38 (0.68, 2.82) | 1.27 (0.57, 2.82) | 1.08 (0.51, 2.29) |
|  | 27-64 years | No change | ref | ref | ref | ref |
|  |  | Better, no worse | **1.35 (1.06, 1.72)** | 1.15 (0.91, 1.45) | **1.29 (1.06, 1.57)** | **1.35 (1.06, 1.71)** |
|  |  | Mixed | **3.31 (2.32, 4.71) ^#^** | **2.13 (1.49, 3.04) ^#^** | **2.56 (1.80, 3.65) ^#^** | **2.05 (1.43, 2.93) ^#^** |
|  |  | Worse, no better | **4.12 (3.15, 5.38) ^#^** | **3.34 (2.57, 4.34) ^#^** | **3.13 (2.40, 4.09) ^#^** | **2.64 (2.03, 3.45) ^#^** |
|  | ≥65 years | No change | ref | ref | ref | ref |
|  |  | Better, no worse | 1.68 (0.92, 3.05) | 1.39 (0.83, 2.33) | 1.22 (0.86, 1.74) | 1.35 (0.79, 2.30) |
|  |  | Mixed | **3.60 (1.38, 9.39)** | 2.19 (0.81, 5.94) | **3.38 (1.65, 6.92)** | **5.59 (2.45, 12.75) ^#^** |
|  |  | Worse, no better | **4.55 (2.41, 8.61) ^#^** | **3.39 (1.91, 6.01) ^#^** | **3.10 (1.97, 4.86) ^#^** | 1.59 (0.81, 3.15) |
|  | *P interaction* |  | *0.32* | *0.15* | *0.18* | *0.32* |
| **Race/ethnicity** | NHW | No change | ref | ref | ref | ref |
|  |  | Better, no worse | **1.43 (1.13, 1.81)** | 1.14 (0.91, 1.43) | 1.20 (1.00, 1.45) | **1.33 (1.06, 1.66)** |
|  |  | Mixed | **3.26 (2.28, 4.66) ^#^** | **2.10 (1.46, 3.01) ^#^** | **2.51 (1.80, 3.52) ^#^** | **2.38 (1.67, 3.39) ^#^** |
|  |  | Worse, no better | **4.19 (3.26, 5.40) ^#^** | **3.20 (2.51, 4.09) ^#^** | **3.10 (2.45, 3.93) ^#^** | **2.13 (1.65, 2.75) ^#^** |
|  | Non-White | No change | ref | ref | ref | ref |
|  |  | Better, no worse | 1.06 (0.66, 1.70) | 1.05 (0.68, 1.62) | 1.06 (0.72, 1.55) | 1.32 (0.83, 2.09) |
|  |  | Mixed | **4.25 (2.07, 8.69) ^#^** | **2.16 (1.08, 4.34)** | **3.68 (1.71, 7.94)** | **2.73 (1.33, 5.61)** |
|  |  | Worse, no better | **2.87 (1.62, 5.08) ^#^** | **2.97 (1.72, 5.13) ^#^** | **2.31 (1.32, 4.05)** | **3.25 (1.86, 5.69) ^#^** |
|  | *P interaction* |  | *0.29* | *0.69* | *0.35* | *0.41* |
| **Sex** | Male | No change | ref | ref | ref | ref |
|  |  | Better, no worse | 1.69 (0.96, 2.97) | 1.46 (0.88, 2.43) | 1.45 (1.00, 2.11) | 1.47 (0.86, 2.50) |
|  |  | Mixed | **10.57 (4.45, 25.13) ^#^** | **6.29 (2.76, 14.31) ^#^** | **4.82 (2.11, 11.05) ^#^** | **8.28 (3.66, 18.73) ^#^** |
|  |  | Worse, no better | **6.50 (3.56, 11.87) ^#^** | **5.08 (2.94, 8.79) ^#^** | **2.98 (1.84, 4.84) ^#^** | **2.32 (1.28, 4.19)** |
|  | Female | No change | ref | ref | ref | ref |
|  |  | Better, no worse | **1.33 (1.06, 1.67)** | 1.07 (0.86, 1.33) | 1.11 (0.93, 1.34) | **1.31 (1.05, 1.64)** |
|  |  | Mixed | **2.97 (2.11, 4.17) ^#^** | **1.76 (1.25, 2.49)** | **2.37 (1.70, 3.29) ^#^** | **1.99 (1.41, 2.81) ^#^** |
|  |  | Worse, no better | **3.68 (2.86, 4.74) ^#^** | **2.87 (2.25, 3.67) ^#^** | **2.97 (2.32, 3.79) ^#^** | **2.25 (1.75, 2.89) ^#^** |
|  | *P interaction* |  | *0.15* | ***0.02*** | *0.73* | *0.47* |

Abbreviations: GAD-7, Generalized Anxiety Disorder Scale-7 items; PHQ-9, Patient Health Questionnaire-9 items; PSS-4, Perceived Stress Scale 4; PC-PTSD, Primary Care PTSD screen; PTSD, Post-traumatic stress disorder; NHW, Non-Hispanic White.

^a^ Adjusted for age, sex, race/ethnicity, education, annual household income, marital status, employment status, and quarantine status.

Bold indicates *P* <0.05 prior to Bonferroni correction. ^#^ indicates *P* <0.05 after Bonferroni correction.


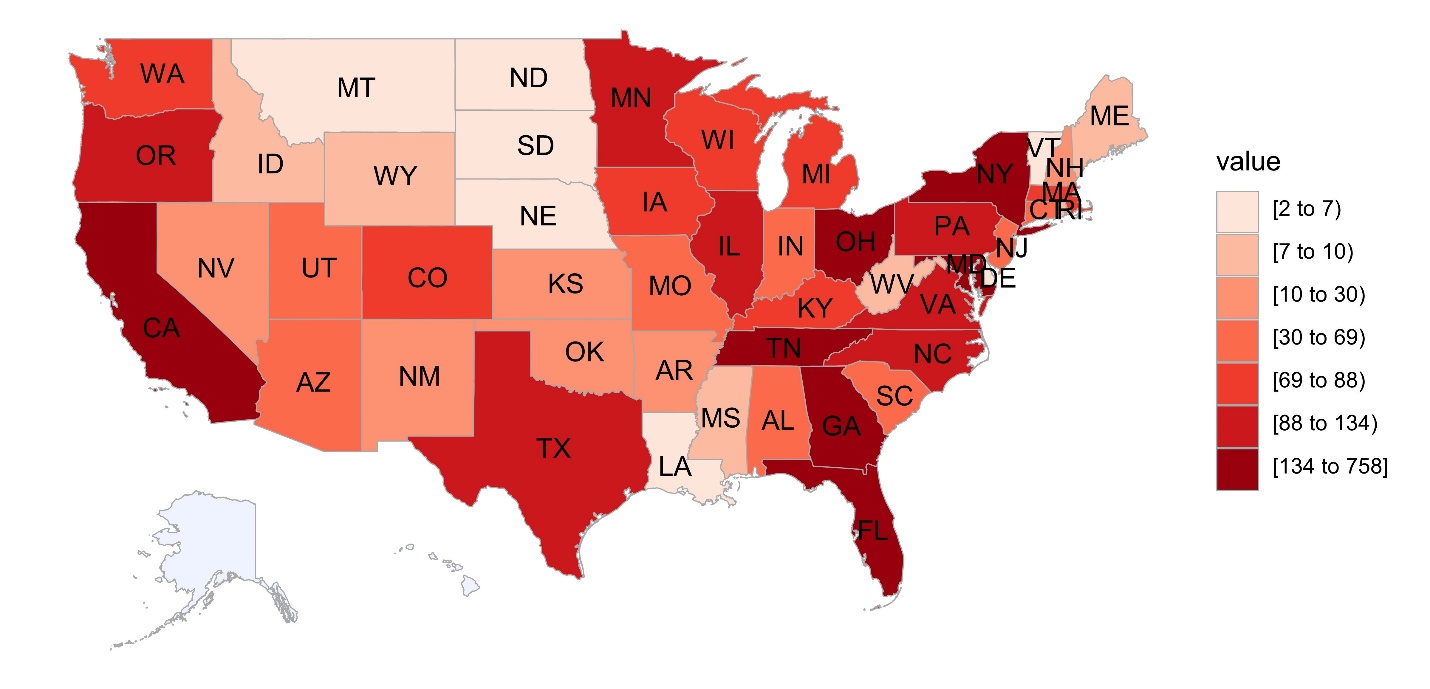


**Figure S1. Geographic distribution of 3,952 participants in this study**

Darker colors represent a larger number of participants in each state.


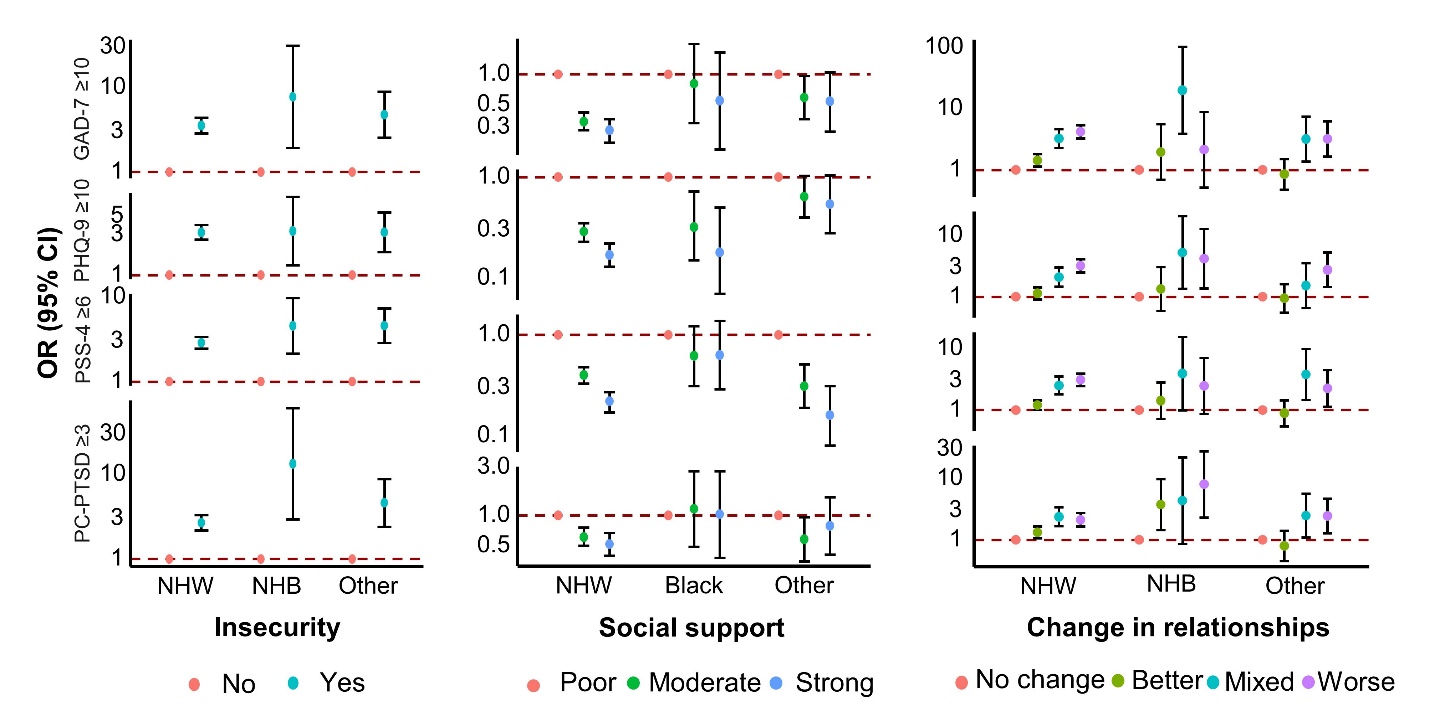


**Figure S2. Associations of insecurity, social support, and change in relationships with indicators of poor mental health stratified by race/ethnicity groups**

Dots represent estimated ORs for symptoms of anxiety (GAD-7 ≥10), depression (PHQ-9 ≥10), stress (PSS-4 ≥6), and trauma-related disorders (PC-PTSD score ≥3), and error bars construct 95% CIs. Dashed lines indicate the null association (OR=1). Abbreviations: GAD-7, Generalized Anxiety Disorder Scale-7 items; PHQ-9, Patient Health Questionnaire-9 items; PSS-4, Perceived Stress Scale 4; PC-PTSD, Primary Care PTSD screen; PTSD, Post-traumatic stress disorder; OR, odds ratio; CI, confidence interval; NHW, Non-Hispanic White; NHB, Non-Hispanic Black. Other race/ethnicity includes participants self-identified as Native American/American Indian, other races, or multi-racial.
